# Anti-tumor effect of bisphosphonate (YM529) on non-small cell lung cancer cell lines

**DOI:** 10.1186/1471-2407-7-8

**Published:** 2007-01-12

**Authors:** Ryuichiro Koshimune, Motoi Aoe, Shinichi Toyooka, Fumikata Hara, Mamoru Ouchida, Masaki Tokumo, Yoshifumi Sano, Hiroshi Date, Nobuyoshi Shimizu

**Affiliations:** 1 Department of Cancer and Thoracic Surgery, Graduate School of Medicine, Dentistry and Pharmaceutical Sciences, Okayama University, 2-5-1 Shikata-cho, Okayama city, Okayama 700-8558, Japan; 2 Department of Molecular Genetics, Graduate School of Medicine, Dentistry and Pharmaceutical Sciences, Okayama University, 2-5-1 Shikata-cho, Okayama city, Okayama 700-8558, Japan

## Abstract

**Background:**

YM529 is a newly developed nitrogen-containing bisphosphonate (BP) classified as a third-generation BP that shows a 100-fold greater potency against bone resorption than pamidronate, a second-generation BP. This agent is, therefore expected to be extremely useful clinically for the treatment of osteoporosis and hypercalcemia. Recently, YM529 as well as other third-generation BPs have also been shown to exert anti-tumor effects against various types of cancer cells both *in vitro *or/and *in vivo*. In this study, we investigate the anti-tumor effect of YM529 on non-small cell lung cancer (NSCLC).

**Methods:**

Direct anti-tumor effect of YM529 against 8 NSCLC cell lines (adenocarcinoma: H23, H1299, NCI-H1819, NCI-H2009, H44, A549, adenosquamous cell carcinoma: NCI-H125, squamous cell carcinoma: NCI-H157) were measured by MTS assay and calculated inhibition concentration 50 % (IC_50_) values. YM529 induced apoptosis of NCI-H1819 was examined by DNA fragmentation of 2 % agarose gel electrophoresis and flowcytometric analysis (sub-G_1 _method). We examined where YM529 given effect to apoptosis of NSCLC cells in signaling pathway of the mevalonate pathway by western blotting analysis.

**Results:**

We found that there was direct anti-tumor effect of YM529 on 8 NSCLC cell lines in a dose-dependent manner and their IC_50 _values were 2.1 to 7.9 μM and YM529 induced apoptosis and G_1 _arrest cell cycle with dose-dependent manner and YM529 caused down regulation of phospholyration of ERK1/2 in signaling pathways of NSCLC cell line (NCI-H1819).

**Conclusion:**

Our study demonstrate that YM529 showed direct anti-tumor effect on NSCLC cell lines in vitro, which supports the possibility that third-generation BPs including YM529 can be one of therapeutic options for NSCLC.

## Background

Lung cancer is the leading cause of death from cancer worldwide. Approximately 80% of lung cancers can be histologically classified as non-small cell lung cancers (NSCLCs). The majority of patients presents with locally advanced (37%) or metastatic (38%) disease at the time of diagnosis [1]. Despite advances in chemotherapy, the average 5-year survival rate of the patients with advanced NSCLC remains extremely poor [1]. Thus, newer agents must be developed to establish an effective therapeutic strategy against NSCLC.

Bisphosphonates (BPs) are structural analogues of pyrophosphoric acid, which is a biomedical component. Several studies have shown that BPs exert direct anti-tumor effects on a variety of human tumor cell lines (myeloma, breast, prostate, pancreas) *in vitro *in a concentration- and time-dependent manner [2-13]. BPs have a common basic structure and different substituents at the one of two covalentlybounded side-chain attached to the germinal carbon, which strongly influence their pharmacologic properties [14]. The so-called second- or third-generation BPs contain nitrogen and inhibit protein prenylation in osteoclasts to induce apoptosis [14-17]. YM529 (1-Hydroxy-2-imidazo [1,2-a]pyridin-3-yl) ethylidene bisphosphonic acid monohydrate is a newly developed nitrogen-containing BP classified as a third-generation BP that shows a 100-fold greater potency against bone resorption than pamidronate, a second-generation BP. This agent is, therefore expected to be extremely useful clinically for the treatment of osteoporosis and hypercalcemia.

Recently, YM529 as well as other third-generation BPs have also been shown to exert anti-tumor effects against various types of cancer cells both *in vitro *or/and *in vivo *[7,13,18-22]. However, study that evaluated the effect of BPs including YM529 on NSCLC has been limited and the effect of zoledronic acid on only one cell line was examined [23].

In this study, we examined the effects of YM529 on 8 NSCLC cell lines to investigate the potential usefulness of YM529 as a therapeutic agent against NSCLC.

## Methods

### Reagents and cell lines

YM529 was kindly provided by Astellas Pharmaceuticals Co., Ltd. (Tokyo, Japan). YM529 was diluted in 10 mM of concentration and stored at -20°C for *in vitro *experiment.

Eight human NSCLC cell lines (adenocarcinoma; NCI-H23, NCI-H1819, NCI-H2009, HCC44, A549, squamous cell carcinoma; NCI-H157, adenosquamous cell carcinoma; NCI-H125, large cell carcinoma; NCI-H1299, there are involved all type of NSCLC) were kindly provided by Dr. Adi F. Gazdar (University of Texas Southwestern Medical Center, Dallas, USA). All 8 cell lines were maintained at 37°C in a fully humidified atmosphere of 5% CO_2 _in air as suspension cultures in RPMI-1640 medium (Sigma chemical co. St. Louis, MO) supplemented with 10% fetal bovine serum (FBS) and 100 U/ml penicillin and 100 μg/ml streptomycin (Sigma chemical co. St. Louis, MO).

### Determination of cell proliferation

Cell proliferation was determined by a modified MTS assay with CellTiter 96^® ^AQueous One Solution Reagent (Promega, Madison, WI). NSCLC cells were seeded on 96-well flat-bottomed tissue culture plates(Becton Dickinson, San Jose, CA) at a concentration of 5 × 10^3 ^cells/well with complete culture medium and allowed to adhere to the plate overnight. Then the cells were incubated in the presence of each concentration of 0(control), 0.01, 0.1, 1, 5, 10, 20, 50, 100, 200 μM of YM529 for another 72 hours at 37°C in a humidified atmosphere of 5% CO_2 _in air. After treatment, 20 μl of CellTiter 96^® ^AQueous One Solution Reagent were dropped into each well of plates. After 90 minutes incubation, the optical densities (OD) of these samples were directly measured using an Immuno Mini NJ-2300 (Nalge Nunc International KK, NY, USA) and reference wavelength of 490 nm. The OD of control samples was regarded as 100. Each condition was performed with 8 wells and each experiment was repeated twice. The anti-proliferative activities of YM529 are shown in terms of IC_50_s.

### Electrophoresis of DNA fragment

NCI-H1819 cells were treated with concentrations 0 (negative control) and 10, 100 μM of YM529 for 48 hours. As positive control, NCI-H1819 was incubated for 24 hours after UV light (10 mJ/cm^2^) irradiation. Genomic DNA was isolated by digestion with proteinase K followed by phenol:chloroform (1:1) extraction and ethanol precipitation from cell lines and primary tumor [24].

DNA and DNA marker of TrackIt 1 kb Plus Ladder (Invitrogen co. Carlsbad, CA, USA) were separated on 2 % agarose gels.

### Flow cytometric analysis of apoptosis

Subconfluent NCI-H1819 cells were cultured in 60 mm dishes with concentrations 0(control), 10 and 100 μM of YM529 for 24 hours. Cells were trypsinized, fixed in 70 % iced-cold ethanol, and stored at -20°C for 72 hours. After fixation, cells were suspended in 100 μl phosphate-citrate buffer (0.19 M Na_2_HPO_4_, 4 mM citric acid) and incubated for 30 min at room temperature and resuspended in 1 ml of PBS containing 10 μg/ml of propidium iodide (PI) and 10 μg/ml of RNase A. The PI-stained cell samples were analyzed using FACSCalibur (Becton-Dickinson San Jose, CA) and data analysis for the population of cells in sub-G_1_, G_1_, S and G_2_/M phase of the cell cycle was performed with CELLQuest (Becton-Dickinson San Jose, CA). Cells undergoing apoptosis were determined as a percentage of cells with sub-G_1 _population.

### Western blotting analysis

Subconfluent NCI-H1819 cells were cultured in 60 mm dishes with concentrations 0(control), 10 and 100 μM of YM529 for 24 hours. Equal amount of protein (10 μg) were separated by SDS-PAGE and transferred to PVDF membranes. The membranes were probed with monoclonal anti-Ras (Becton Dickinson biosciences, San Jose, CA), goat polyclonal anti-unprenylated Rap1A antibody (Santa Cruz Biotechnology inc., Santa Cruz, CA), anti-ERK1/2 (Sigma chemical co. St. Louis, MO), anti-phospho-ERK1/2, anti-Akt, anti-phospho-Akt, β-actin (Cell Signaling Technology Inc., MA, US) antibodies, and then with goat anti-rabbit, goat anti-mouse and rabbit anti-goat IgG-HRP coupled to horseradish peroxidase conjugated secondary antibodies (Santa Cruz biotechnology inc., Santa Cruz, CA), after which the membranes were developed by ECL Plus Western Blotting Detection Reagents (Amersham Biosciences UK Limited, Buckinghamshire, UK).

## Results

### YM529 inhibited growth of NSCLC cell lines

**Figure 1:**
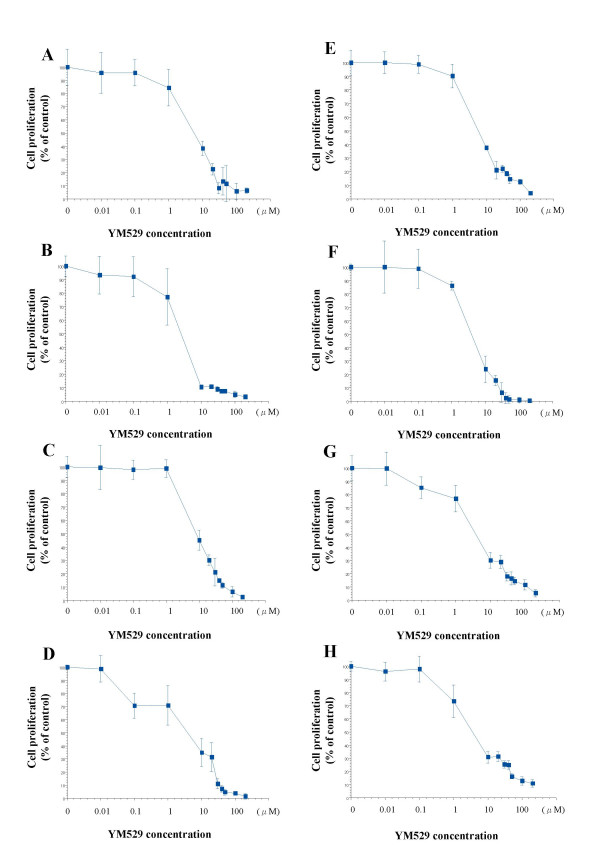
Direct effect of YM529 on 8 cell lines of NSCLC cell lines H23 (A), H1299 (B), NCI-H1819 (C), NCI-H2009 (D), H44 (E), A549 (F), NCI-H125 (G), NCI-H157 (H). Cells were incubated with concentration of 0(control), 0.01, 0.1, 1, 5, 10, 20, 50, 100 μM of YM529 for 72 hours exposure, and cell proliferation was determined by MTS assay. Date represent cell number during treatment relative to control untreated cells (100%). The averages: bars, SD.

To evaluate the growth inhibitory effect of YM529 on NSCLC cell lines, we used the MTS assay and calculated the IC_50 _values. YM529 inhibited cell proliferation in a concentration-dependent manner in all the 8 cell lines (Fig. 1). The IC_50 _values for the 8 NSCLC cell lines ranged from 2.1 to 7.9 μM. Because YM529 inhibited the proliferation of NCI-H1819 most strongly among 8 NSCLC cell lines (Fig. 1), for following experiments, this cell line (NCI-H1819) was used as a model line.

### Induction of apoptosis

**Figure 2:**
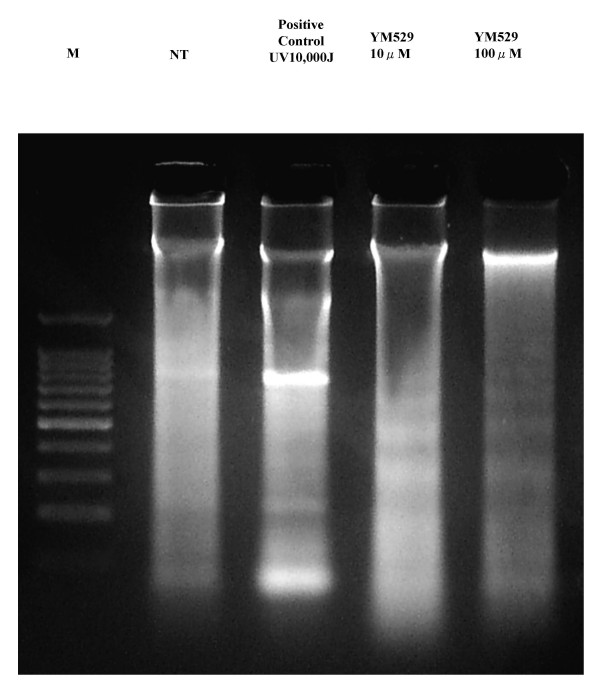
DNA fragmentation induced by treatment of YM529 (10, 100 μM) and UV irradiation. DNA of UV irradiation (10 mJ/cm^2^) was positive control. DNAs of no treatment (negative control), positive control, treatment of 10, 100 μM YM529 were fractionated through a 2 % agarose gel and stained with ethidium bromide. Lane M in panel is size markers.

**Figure 3:**
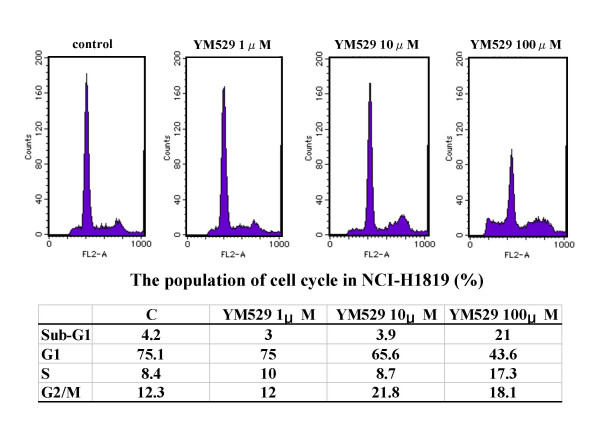
Cell cycle analysis of NSCLC cells treated with YM529. NCI-H1819 cells were stained with PI after 24 hours exposure of no treatment (control), 1, 10 100 μM of YM529. There were analyzed by flow cytometry. DNA histgram of control (A), YM529 1 μM (B), YM529 10 μM (C), YM529 100 μM (D) were shown. The percentages of the cell population in the different phase (sub-G_1_, G_1_, S, G_2_/M) of cell cycle were analyzed by CELLQuest. Percentages of apoptosis were measured as sub-G_1 _content of histogram. Similar results were obtained in three independent experiments.

To confirm that YM529 caused apoptosis, we examined the fragmentation status of genomic DNA in NCI-H1819 cells treated with drug (Fig. 2). DNA was extracted and electorophoresis was performed. As shown in Fig. 3, short fragments of DNA were observed in the cell cultures treated with 10 and 100 μM of YM529, similar to the result in the positive control which was represented by NCI-H1819 cells exposed to UV light irradiation at 10 mJ/cm^2 ^(Fig. 2).

### Cell cycle distribution

To elucidate the molecular mechanism underlying the anti-tumor activity of YM529, we examined the cell cycle distribution in NCI-H1819 cells by flow cytometric analysis after PI staining. No significant changes in the S and G_2_/M phase of the cell cycle was observed for any concentration of YM529. However, an increase in the percentage of cells in the G_1 _phase was observed in a YM529-concentration-dependent manner (Fig. 3). While there were no significant changes in the population of cells in the sub-G_1 _phase in the cells subjected to no treatment or treated with 1 or 10 μM of YM529 (4.2, 3.0, and 3.9 %, respectively), a significant increase in the population of cells in the sub- G_1 _phase was observed in the cells treated with 100 μM of YM529 (21.0%) (Fig. 3). These results suggest that YM529 induced cell cycle arrest at the G_1 _phase and consequently stimulated apoptosis in the NCI-H1819 cell line.

### Down-regulation of anti-apoptosis

**Figure 4:**
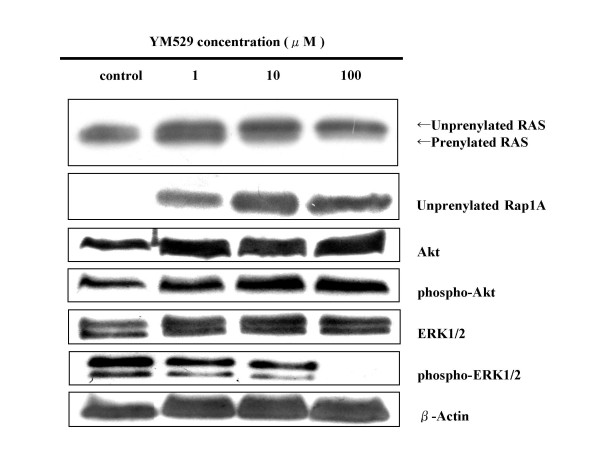
Concentration- course changes of Ras, Rap1A, Akt, phospho-Akt, ERK1/2, phospho-ERK1/2 activation in NCI-H1819 cells treated with YM529 by using Western blot. Cells were incubated in CM with 10 % FBS. Equal amounts of protein were loaded in each lane. β-Actin was used as internal control.

To identify the molecules involved in the YM529-induced cell cycle arrest and apoptosis, we examined the protein expression status of members of the small GTP-binding protein related cascade that YM529 was assumed to inhibit in order to cause apoptosis. Western blotting analysis showed that anti-Ras or anti-unprenylated Rap1A antibody recongnized the unprenylation of Ras or Rap1A in a dose-dependent manner of YM529 (Fig. 4). In addition, the phospho-ERK1/2 protein was down-regulated by YM529 treatment. (Fig. 4). These results suggested that YM529 inhibited ERK1/2 pathway via inhibition of farnesylation and/or geranylgeranylation of GTP-binding proteins. There were no significant changes in the expression of either Akt or phospho-Akt in the cells.

## Discussion

Our studies showed that YM529 directly inhibited cell proliferation in NSCLC cell lines. The IC_50 _values of YM529 for the 8 cell lines examined ranged from 2.1 to 7.9 μM. While the effects of third-generation BPs, including YM529, YM175 and zoledronic acid, have been examined in several kinds of human cancers, this is the first study to shown that the third-generation BPs may also be effective against NSCLC [9-13,22,23]. YM529 is as potent as zoledronic acid at inhibiting bone resorption *in vivo*, despite the difference in the substituent at the covalentlybounded side-chain. In relation to the effects of these agents on human cancer, YM529 and zoledronic acid have been reported to show similar cytotoxic effects against luekemic cell lines, with an IC_50 _value of 22–73 μM [19], which also indicates that the IC_50 _value of YM529 for NSCLC was lower than that for leukemic cell lines. Indeed, the IC_50 _value for leukemic cells in our study was similar to that reported previously, which also indicated that our assay was appropriate. In the case of lung cancer, the effect of zoledronic acid was examined against 12 small-cell lung cancer (SCLC) cell lines; it was shown that the agent exerted anti-proliferative effect in of the 8 cell lines with IC_50 _values in the range of 13–30 μM, whereas the remaining cell lines were resistant to zoledronic acid [22]. Taking into account this finding with our own, it appears that third-generation BPs may be more effective against NSCLC than against SCLC.

In regard to the mechanism of action of nitrogen-containing BPs, including YM529, it is thought that they inhibit the activity of farnesyl diphosphate (FPP) synthase and geranylgeranyl diphosphate (GGPP) synthase which cause activation of FPP and GGPP, respectively. Since FPP and GGPP activation cause prenylation of small GTP-binding proteins including Ras resulting in anti-apoptosis, inhibition of their activation by YM529 induces cellular apoptosis [25-29]. Indeed, YM529 induced unprenylation of Ras and Rap1A resulting in down-regulation of ERK1/2 phosphorylation in NCI-H1819 cells despite absence of any effect on ERK1/2 expression. Recent reports have shown that inhibition of the ERK1/2 pathway reduces cycline-CDK2 kinase activity and subsequent G_1 _arrest indicating apoptosis [30,31]. Our flow cytometirc analysis of the NCI-H1819 cell line confirmed the increase of the G_1 _population with YM529 treatment. Taking together these findings, it is speculated that YM529 exerts its anti-proliferative effect against NSCLC by induction of cellular apoptosis through the small GTP-binding proteins associated signal transduction pathway.

Several studies have indicated the usefulness of the combined use of BPs with other cytotoxic agents [12,22,32]. Matsumoto et al. reported that zoledronic acid augmented the anti-proliferative activity of paclitaxel, etoposide, cisplatinum, irinotecan and imatinib in SCLC cells [22]. In addition, cytotoxic agents, including cisplatin, gemcitabine, and taxol shown enhanced anti-tumor effect when combined with a farnesyltransferase inhibitor (FTI-2148) or geranylgeranyltransferase inhibitor (GGTI-2154) in mice bearing A549 cells [32]. Because YM529 is assumed to inhibit farnesylation and geranylgeranylation synthase as mentioned, combined treatment with YM529 and these cytotoxic agents may be a more effective treatment strategy than monotherapy in the case of NSCLC as well.

## Conclusion

Our study demonstrated that YM529 showed direct anti-tumor effect on NSCLC cell lines in vitro and induced apoptosis and G_1 _arrest cell cycle through down regulation of phospholyration of ERK1/2. These findings support the possibility that third-generation BPs including YM529 can be one of therapeutic options for NSCLC.

## Competing interests

The author(s) declare that they have no competing interests.

## Authors' contributions

RK drafted the manuscript and carried out the experiment. MA conceived the study. ST managed the experiment and edited the manuscript. FH carried out western blotting. MO and MT carried out extracting DNA and electorophoresis for DNA. YS gave valuable advice for flow cytometric assay. HD and NS conceived the study and participated in its coordination. All authors read and approved this manuscript.

## Pre-publication history

The pre-publication history for this paper can be accessed here:
